# Supplementary material for: DMRforPairs: identifying Differentially Methylated Regions between unique samples using array based methylation profiles
Source: BMC Bioinformatics. 2014 May 15;15:141. doi: 10.1186/1471-2105-15-141 (PMC4046028; doi:10.1186/1471-2105-15-141)
Supplement: Additional file 2 — DMRforPairs output for the comparison of A431-MCF7 and NA17018-NA17105. Please start from the HTML files in each folder. Available via the BMC Bioinformatics website. [file 1471-2105-15-141-S2.zip › 1394847754114233_MOESM2_ESM/A431_MCF7/figures/27191.pdf]

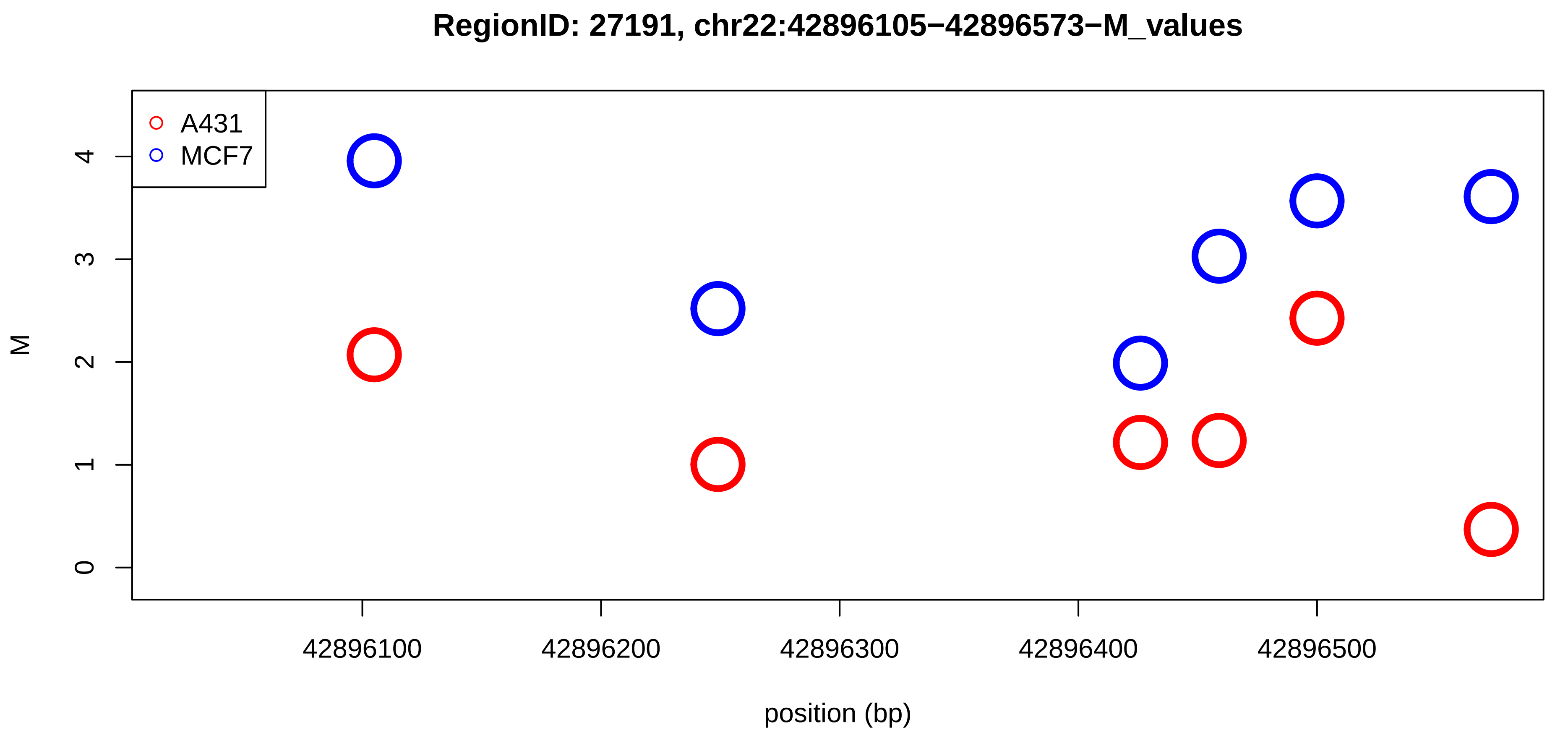

RegionID: 27191, chr22:42896105−42896573−M_values
A431
MCF7
M
0
1
2
3
4
42896100
42896200
42896300
42896400
42896500
position (bp)

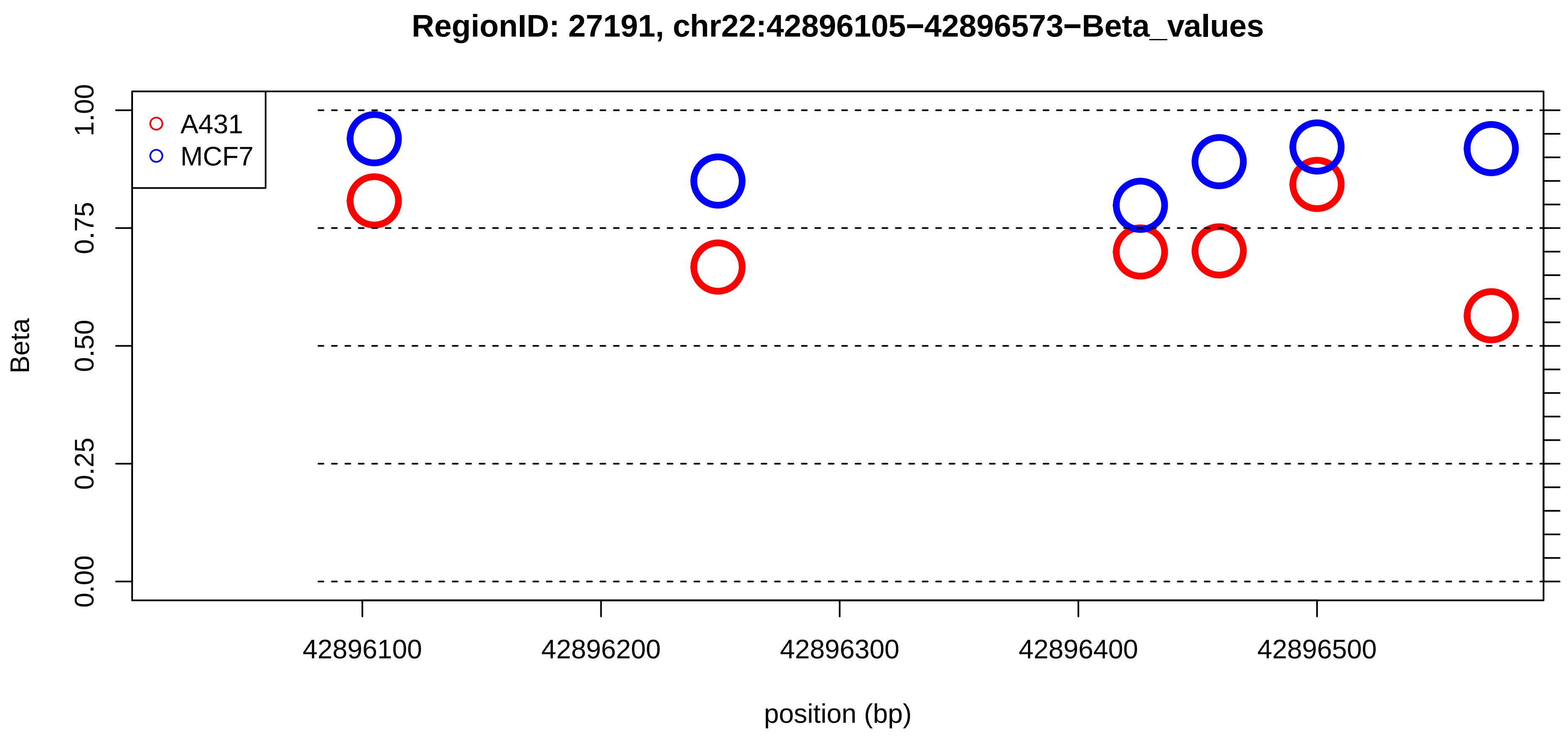

RegionID: 27191, chr22:42896105−42896573−Beta_values
A431
MCF7
Beta
0.00
0.25
0.50
0.75
1.00
42896100
42896200
42896300
42896400
42896500
position (bp)

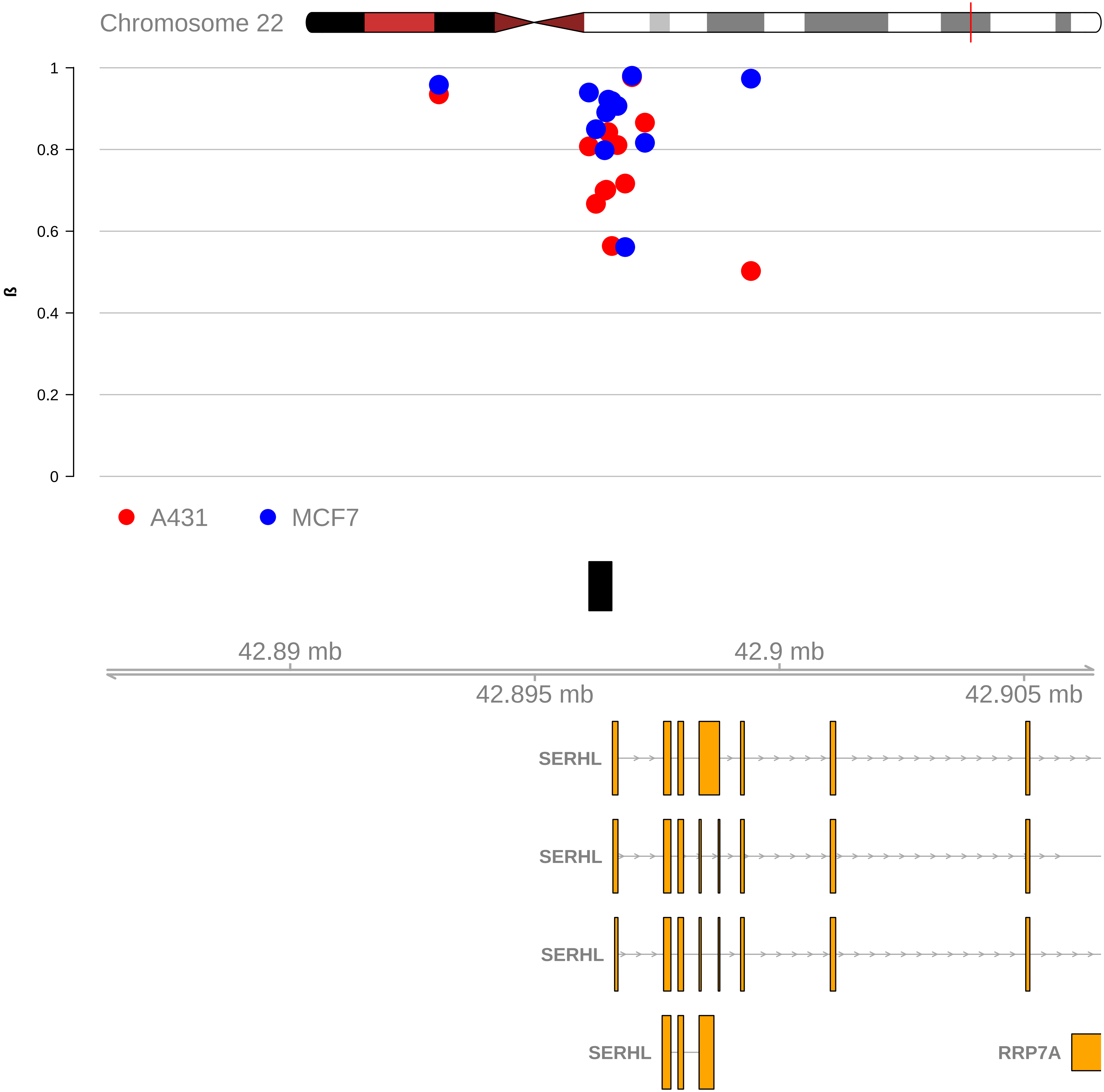

Chromosome 22
ß
1
0.8
0.6
0.4
0.2
0
A431
MCF7
42.89 mb
42.9 mb
42.895 mb
42.905 mb
SERHL
SERHL
SERHL
SERHL
RRP7A
